# The essentiality landscape of cell cycle related genes in human pluripotent and cancer cells

**DOI:** 10.1186/s13008-019-0058-4

**Published:** 2019-12-23

**Authors:** Ruth Viner-Breuer, Atilgan Yilmaz, Nissim Benvenisty, Michal Goldberg

**Affiliations:** 1 The Azrieli Center for Stem Cells and Genetic Research, The Hebrew University, Givat-Ram, 9190401 Jerusalem, Israel; 2 Department of Genetics, Institute of Life Sciences, The Hebrew University, Givat-Ram, 9190401 Jerusalem, Israel

**Keywords:** Cell cycle, CRISPR/Cas9 libraries, Embryonic stem cells, Cancer, *TP53*, Checkpoints, Bioinformatics

## Abstract

**Background:**

Cell cycle regulation is a complex system consisting of growth-promoting and growth-restricting mechanisms, whose coordinated activity is vital for proper division and propagation. Alterations in this regulation may lead to uncontrolled proliferation and genomic instability, triggering carcinogenesis. Here, we conducted a comprehensive bioinformatic analysis of cell cycle-related genes using data from CRISPR/Cas9 loss-of-function screens performed in four cancer cell lines and in human embryonic stem cells (hESCs).

**Results:**

Cell cycle genes, and in particular S phase and checkpoint genes, are highly essential for the growth of cancer and pluripotent cells. However, checkpoint genes are also found to underlie the differences between the cell cycle features of these cell types. Interestingly, while growth-promoting cell cycle genes overlap considerably between cancer and stem cells, growth-restricting cell cycle genes are completely distinct. Moreover, growth-restricting genes are consistently less frequent in cancer cells than in hESCs. Here we show that most of these genes are regulated by the tumor suppressor gene *TP53*, which is mutated in most cancer cells. Therefore, the growth-restriction system in cancer cells lacks important factors and does not function properly. Intriguingly, M phase genes are specifically essential for the growth of hESCs and are highly abundant among hESC-enriched genes.

**Conclusions:**

Our results highlight the differences in cell cycle regulation between cell types and emphasize the importance of conducting cell cycle studies in cells with intact genomes, in order to obtain an authentic representation of the genetic features of the cell cycle.

## Background

The cell cycle is the process of growth and proliferation of living cells, during which each cell replicates its genome and divides into two daughter cells. This is a highly complex and organized process that consists of 4 consecutive phases: G1, S, G2 and M, and requires the scheduled occurrence of a large series of events [1]. Naturally, faithful execution of the cell cycle is of utmost importance, and many layers of regulation have evolved to ensure its integrity. The most prominent regulation layer is the periodic expression of cyclins, which allows the ordered activation of specific cyclin-dependent-kinases (CDKs), which, in turn, regulate the transition of the cells through cell cycle phases [2]. Another regulatory layer is embodied by the cell cycle checkpoint mechanisms. Checkpoints are control mechanisms activated at different points along the cell cycle to monitor its integrity and fidelity by allowing cell cycle progression only under satisfying conditions. For example, DNA damage can trigger checkpoint activation at the G1/S phase and the G2/M transition points as well as during the S phase (the intra-S checkpoint), thus preventing the transmission of DNA alterations to the newly formed cells. Depending on the extent of the damage, the checkpoints promote either DNA repair, or, if the damage is too excessive, apoptosis or senescence of the affected cells [3, 4]. Additional triggers for checkpoint activation are chromosomes that are unattached or improperly attached to the opposite spindle poles during mitosis. These may lead to the activation of the M phase spindle assembly checkpoint that prevents unequal inheritance of the genetic material. Impairment in checkpoint mechanisms may cause genomic instability, leading to severe phenotypes, such as tumorigenesis, developmental delay and intellectual disability [5–7].

There is a close relationship between cell cycle regulation and cancer etiology. Cancer cells are characterized by genomic instability; they possess the capacity for unlimited cell divisions, and are characterized by an uncontrolled cycle, which can progress independently of growth signals. Abrogated activity of cell cycle factors, such as CDKs and checkpoint proteins, is highly frequent in cancer cells; and such mutations in cell cycle genes are often associated with tumorigenesis [7–9].

Moreover, genes participating in the inhibition of CDKs often act as tumor suppressors, and some of them are regulated by p53, which is encoded by the *TP53* gene and promotes apoptosis in response to DNA damage, mainly through the G1/S checkpoint. *TP53* is referred to as the “guardian of the genome” since it is the most prominent tumor suppressor protein and the most mutated gene in human cancers [8, 10].

Like cancer cells, pluripotent stem cells, such as embryonic stem cells (ESCs), are capable of unlimited proliferation; but, unlike cancer cells, they have differentiation capacity into various cell types, a feature that is retained through infinite cell divisions, by the process of self-renewal. The cell cycle machinery was shown to be tightly associated with pluripotency state, since abrogated activity of cell cycle components affects pluripotency and vice versa [9]. The cell cycle in ESCs has distinct features compared to differentiated and cancerous cells. These include fast proliferation, shortened G1 and G2 phases and a relatively high percentage of cells in S phase [11, 12]. Accordingly, cells that are committed to differentiation undergo many cell cycle changes including the lengthening of G1 phase [13]. These alterations appear to be the cause of cell fate decisions, since cell cycle machinery is actively involved in the determination of the pluripotency state. The short G1 phase of ESCs was shown to disrupt the formation of 53BP1 nuclear bodies around chromosomal lesions, preventing their protective effect against erosion, thus causing a replication stress in the next S phase [14]. Nevertheless, this shortening also appears to have a positive role in pluripotency maintenance. Generally, G1 is considered to be the most important phase in the context of stem cell fate decisions, as in this phase CDKs are regulating the activation of developmental genes, which respond to differentiation signals. This activation initiates the differentiation cascade, a transcriptional program that ultimately leads to cell fate changes [13]. Notably, different CDKs can activate diverse targets, leading to distinct lineage differentiation events [12]. Contrary to G1 phase, it appears that S and G2 phases actively, and independently of G1, support the maintenance of the pluripotent state [15].

The fast proliferation and short cycle of stem cells lead to high frequency of DNA lesions, since the short G1 phase does not leave enough time for the repair of nonreplicated DNA and therefore risks the quality of subsequent DNA replication. Furthermore, these cells are reported to have impaired activation of the G1/S checkpoint upon DNA damage [16]. However, the acquired lesions encounter a fortified wall of robust and constitutively active DNA damage response that efficiently deals with the damage and maintains a relatively low mutation frequency [17, 18]. Interestingly, a previous study which identified the essential genes for the normal growth and survival of human pluripotent stem cells, demonstrated that more than 50% of the essential and transcriptionally enriched genes in these cells were involved in the cell cycle and DNA repair processes [19, 20].

In this study, we analyzed the genetic networks underlying general and unique cell cycle traits, by identifying genes that have common and unique functional impact on the proliferation and survival of cancer and embryonic stem cells. We found that genes linked to S phase and to the checkpoint mechanisms are particularly essential for the proliferation of both cell types. However, the differences observed between the cell cycle essentialomes of pluripotent and cancer cells were largely based on differential essentiality of checkpoint genes between these cell types. In addition, we identified specific cell cycle genes that may play a role in the different properties of each cell type and illuminated a selective dependency of pluripotent cells on the proper function of the spindle assembly checkpoint mechanism. Notably, we found great differences in the genetic networks responsible for growth restriction between pluripotent stem cells and cancer cells.

## Results

### Differences in growth dependency of cell cycle genes between cancer and pluripotent cells

**Fig. 1:**
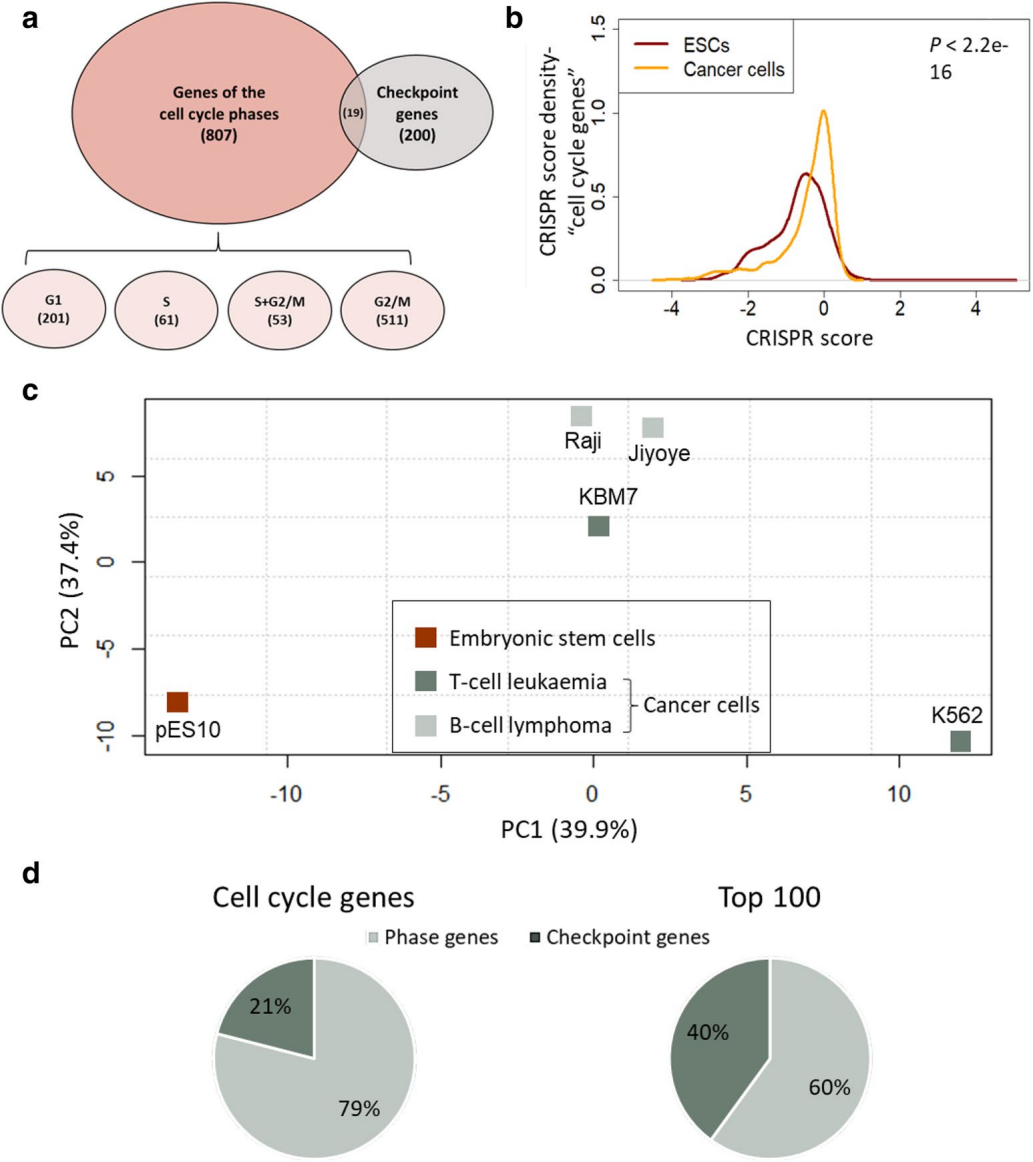
Cell cycle genes have distinct essentiality signatures in pluripotent and cancer cells. **a** Venn diagram showing the overlap of cell cycle gene sets examined in this study. **b** CRISPR score distributions of cell cycle genes in ESCs (red curve) and cancer cells (orange curve). CRISPR score per gene for cancer cells represents the average score across four transformed cell lines. P-value of Kruskal Wallis test is shown. **c** PCA plot demonstrating the separation of essentiality scores for cell cycle genes across different cell lines. **d** Fraction of checkpoint genes among all cell cycle genes as compared to their fraction among the top 100 genes contributing to PC1 separation

To shed light on the genetic basis of cell cycle regulation in ESCs and cancer cells, we generated a list of cell cycle genes, consisting of genes of the cell cycle phases and checkpoint genes, retrieved from independent sources (Fig. 1a; Additional file 1: Table S1 and Additional file 2: Table S2). Genes of the cell cycle phases include protein coding genes that have been shown to have a phenotypic effect on the progression of one or more of the phases of the cell cycle [21]. Checkpoint genes are genes involved in cell cycle regulation as well as in the cellular response to DNA damage and the maintenance of genome integrity.

In order to unveil the genetic factors underlying the properties of the cell cycle in the different cell types, we used data from genome-wide CRISPR/Cas9 loss-of-function screens performed in 5 different cell lines: a haploid hESC line (pES10; hereinafter referred as ESC) [19], and four cancer cell lines [22], one of which had a near haploid karyotype. Two of the cancer cell lines originated from T cell leukemia and two from B cell lymphoma, and all four lines had a mutation in *TP53*. All analyzed screens were based on the same single guide RNAs (sgRNAs) library, a feature that was shown to be necessary for a reliable comparison [19]. We chose to analyze several cancer cell lines in order to eliminate the background noise that may stem from the genetic variation between different tumors. The CRISPR/Cas9 screens mentioned above were previously used for mapping the essential and growth-restricting genes in each cell line [19, 22], by comparing the prevalence of each sgRNA immediately following library generation to that of a later time point after several weeks of culturing, and computing a CRISPR score that represents the log2 of the ratio between the final and initial frequencies. A negative score indicates a perturbation in an essential gene for the proper growth and survival of the cells and a positive score implies a perturbation in a growth-restricting gene. The term “growth” in this article refers to the phenotype of cell enrichment that can occur due to changes in the rate of proliferation, apoptosis or differentiation [19]. For this study, we used the computed CRISPR scores of cell cycle genes, and analyzed the differences in genetic features between ESCs and cancer cells.

First, we performed a Kruskal-Wallis test comparing the distribution of the CRISPR scores of cell cycle genes in ESCs and in cancer cells and found a significant (*P* < 2.2e−16) difference between these cell types (Fig. 1b). The distribution of ESCs tended more towards the negative X-axis, indicating that more essential CRISPR scores were found in ESCs as compared to cancer cells (Fig. 1b). Accordingly, comparison of CRISPR scores of cell cycle genes between the different cell lines using principle component analysis (PCA) successfully distinguished between the pluripotent cell line and the 4 cancer cell lines (Fig. 1c). In part, this difference may reflect changes in the magnitude of the effect of various cell cycle factors on cell growth following cancerous transformation. As expected, the B cell-derived cell lines Raji and Jiyoye clustered very closely together (Fig. 1c), suggesting a high resemblance. The difference between the two leukemic cell lines may be due to the unique near haploid nature of KBM7 (haploid in the whole genome except for chromosome 8 and a 30 mega base segment on chromosome 15). Importantly, KBM7 cell line resembled the other cancerous cell lines more than the haploid ESC line pES10 (Fig. 1c), reinforcing the fact that the differences seen are related to the transformation status of these cells rather than their ploidy.

To better understand the genetic basis of the observed discrepancy between the analyzed cell lines, we focused on the top 100 genes that contributed most to PC1 (Additional file 3: Table S3). This list was significantly enriched for essential genes for all cell lines as indicated by two population proportion tests (*P* < 0.00001 for all comparisons; Additional file 4: Fig. S1A). This observation reinforces the suggestion that the difference between the cell types is based on genes that are crucial for the growth of the cells, and probably contribute to the unique features of each cell line. Interestingly, the proportion of checkpoint genes out of the top 100 genes was significantly higher than their overall proportion of cell cycle genes (40% vs. 21%, respectively, *P* < 0.00001 in a two population proportions test; Fig. 1d), indicating a major role for checkpoint genes in the differences between cancer and pluripotent cells. The enriched pathways for these top 100 genes compared with all cell cycle genes included chromosome organization, checkpoint regulation and DNA damage response (Additional file 4: Fig. S1B), all of which are pathways known to be impaired in cancer cells [23].

### Cell cycle genes, especially checkpoint and S phase genes, are highly essential for the growth of pluripotent and cancerous cells

**Fig. 2:**
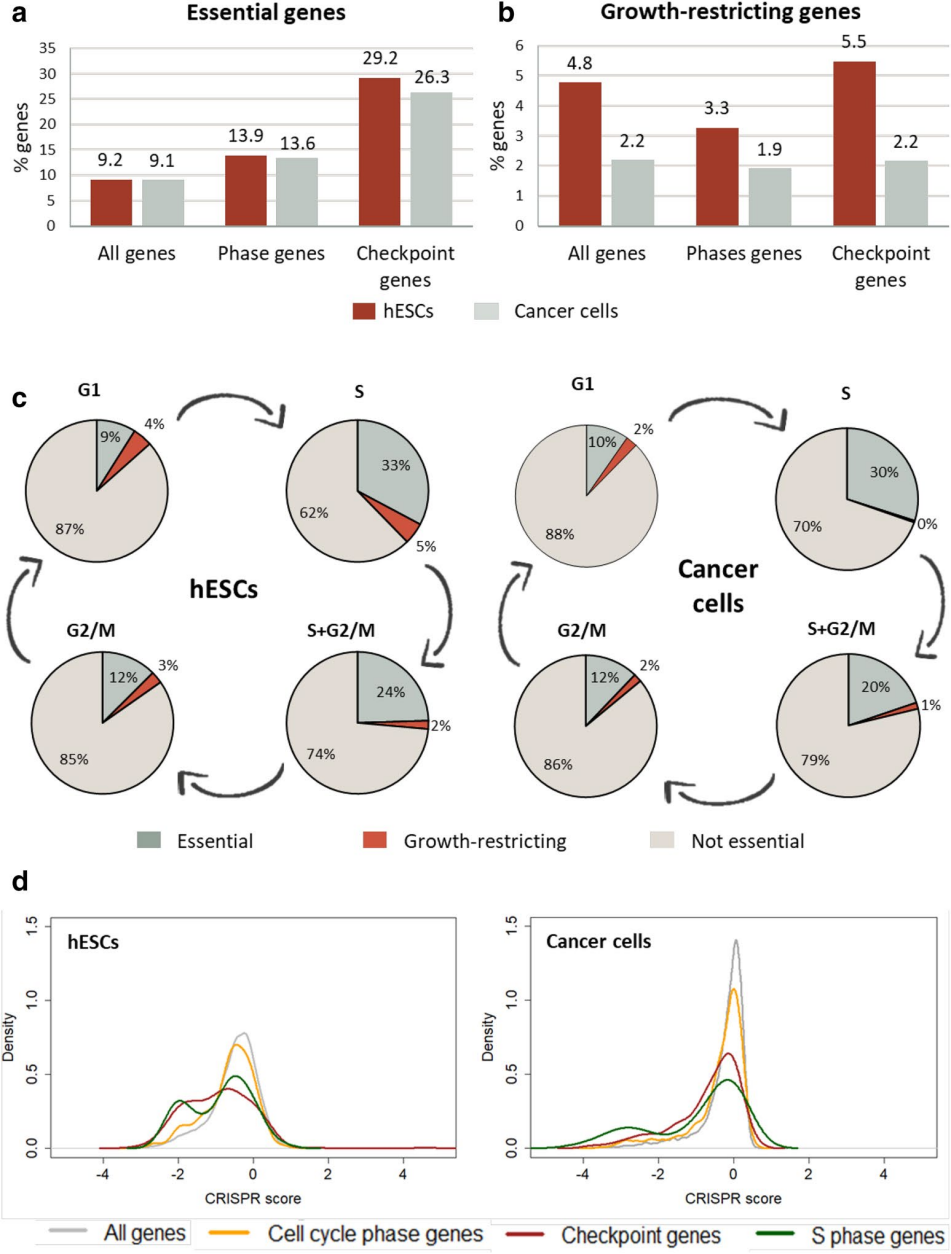
Increased essentiality among checkpoints and S phase genes in both cell types, decreased growth-restriction in cancer. **a** Fractions of essential genes in different gene sets in ESCs (hESCs; red bars) and cancer cells (grey bars). **b** Fractions of growth-restricting genes in different gene sets in ESCs (red bars) and cancer cells (grey bars). **c** Percentages of essential and growth-restricting genes among genes related to different cell cycle phases in ESCs (left) and cancer cells (right). **d** CRISPR score distributions of different gene sets in ESCs (left) and in cancer cells (right). CRISPR score per gene for cancer cells represents the average score across four transformed cell lines

Next, we characterized the essentiality and growth restriction patterns of cell cycle genes in ESCs and in cancerous cells. For each cell line, we used the computed CRISPR scores and classified the genes with FDR < 0.05 as essential for growth or as growth-restricting. Overall, 9.2% of all the protein-coding genes in the human genome were identified as essential for the normal growth of ESCs [19], and 9.1% in average for the growth of cancer cells (Fig. 2a). As expected, genes of cell cycle phases had higher essentiality percentages both in ESCs and in cancer cell lines (Fig. 2a; 13.9% and 13.6%, respectively). Intriguingly, checkpoint genes had an even higher percentage of essentiality both in ESCs and cancer cells (Fig. 2a; 29.2% and 26.3%, respectively). This is in line with the PCA results, in which checkpoint genes were shown to contribute the most to the differences between the CRISPR scores of cell cycle genes in cancer and pluripotent cells.

Conversely, regarding the growth-restricting genes, more genes were identified as growth-restricting in ESCs than in cancer cells (Fig. 2b). Moreover, the percentage of these genes in cancer cells remained low and constant regardless of the gene set examined. This can be due to the fact that cancer cells are often impaired in growth restriction mechanisms, for example because of mutations in tumor suppressor genes [23]. These impairments trigger their rapid proliferation, which is a hallmark of cancer cells. Loss-of-function mutations in genes that take part in the impaired growth restriction pathways would probably not cause an effect on cell cycle progression and therefore they would not be detected in the CRISPR screens as growth-restricting genes.

An interesting observation emerged as we looked at each of the phases separately. Whereas in G1 and G2/M phases the patterns of essentiality did not considerably deviate from the general one, in S phase, and to a lesser extent also in S+G2/M (genes whose downregulation caused a cell cycle arrest in both the S and G2/M phases), the fraction of essential genes was remarkably higher, even higher than that of checkpoint genes. This was true for both ESCs and cancer cells (Fig. 2c; 32.8% and 29.9%, respectively), emphasizing the great importance of regular and accurate operation of all the components of the S-phase regulatory network. In general, the order of essentiality suggested from this analysis in ESCs and cancer cells, is as follows (high to low): S phase genes, checkpoint genes, total cell cycle genes (phases and checkpoints) and all genes (Fig. 2c, d). Notably, the distribution of S phase CRISPR scores seemed to have two peaks, an average one and a very negative one. The latter is likely to represent a group of highly essential s phase genes (Fig. 2d).

### High overlap of essential genes and no overlap of growth-restricting genes among analyzed cell lines

**Fig. 3:**
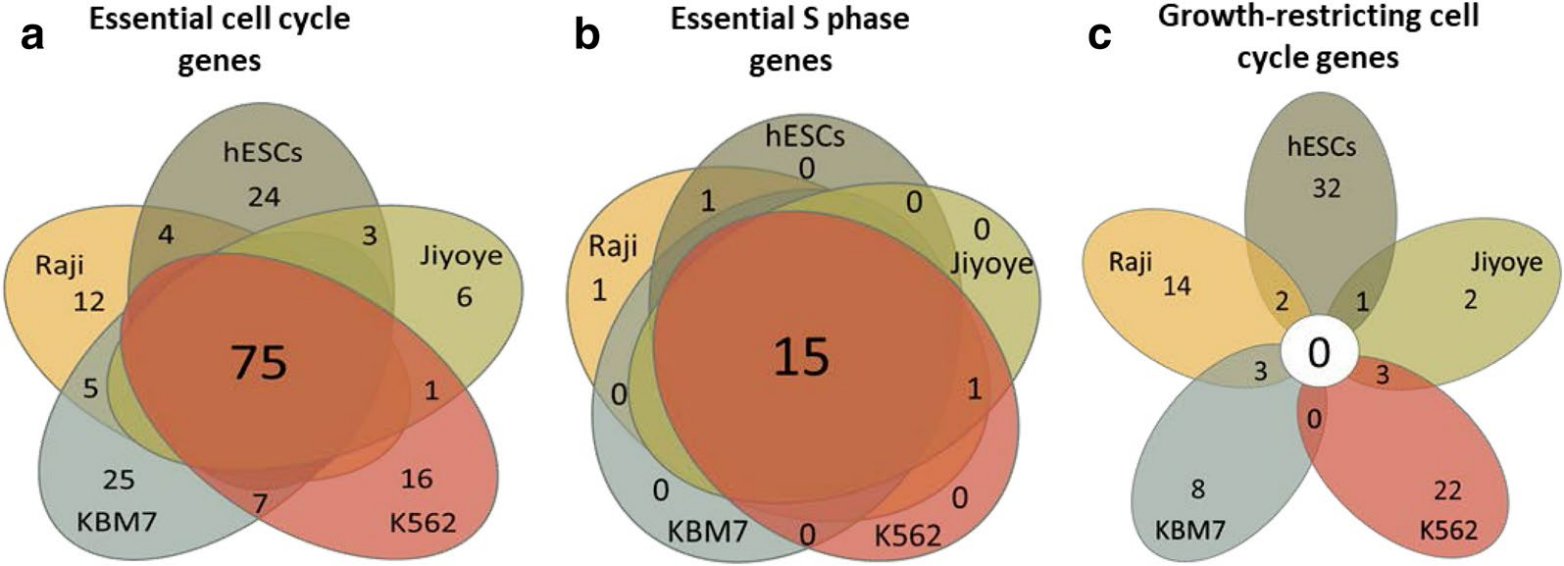
High overlap of essential genes and low overlap of growth-restricting genes among analyzed cell lines. **a**, **b** Venn diagram showing the overlap of essential genes among cell cycle genes (**a**) or S phase genes (**b**) across pluripotent and cancer cells. **c** Venn diagram showing the overlap of growth-restricting genes across pluripotent and cancer cells

To understand whether the same genetic pathways are responsible for cell cycle regulation in different cell types, we checked the degree of overlap between the genes identified as essential or growth-restricting in different cell lines. Regarding the essential genes, there was a considerable overlap between all cell lines, and the highest proportion was that of the common essential genes in all five cell lines (Fig. 3a). Such a large overlap was found both in checkpoint genes and in genes of cell cycle phases (Additional file 4: Fig. S2A), as well as in each phase separately (Additional file 4: Fig. S2B). Arguably, the 75 genes that are common to all 5 cell lines represent the central network of cell cycle regulation that is essential for the growth of all cell types (Additional file 5: Table S4). Interestingly, these genes are involved in a dense network of interactions (Additional file 4: Fig. S3A), in which the most enriched biological process is DNA replication (Additional file 4: Fig. S3B), implying a high proportion of S phase genes. Intriguingly, almost all the essential genes in S phase were common to all 5 cell lines. The number of essential genes in S phase ranged from 16 to 20 in different cell lines, and 15 of them were present in all cell lines (Fig. 3b). This, in addition to the high essentiality of S phase genes (Fig. 2c), further highlights the robustness of S phase and the importance of its integrity for cell survival. It also suggests that this highly conserved group of 15 essential genes represents the “core genes” of S phase, which are essential for cellular growth regardless of the cell type (Additional file 5: Table S4). 7 out of the 15 genes in this list are established DNA replication factors, according to the functional classification in STRING database [24]: CDC6, CDT1, GINS2, POLA1, POLE2, GINS2, RRM1, and RRM2.

Strikingly, when we analyzed the growth-restricting genes, a very different picture emerged. The vast majority of growth-restricting cell cycle genes were unique to each cell line, and not even one gene was common to all cell lines analyzed in this study (Fig. 3c).

### Differential essentiality analysis reveals unique pathways for pluripotent and cancer cells

**Fig. 4:**
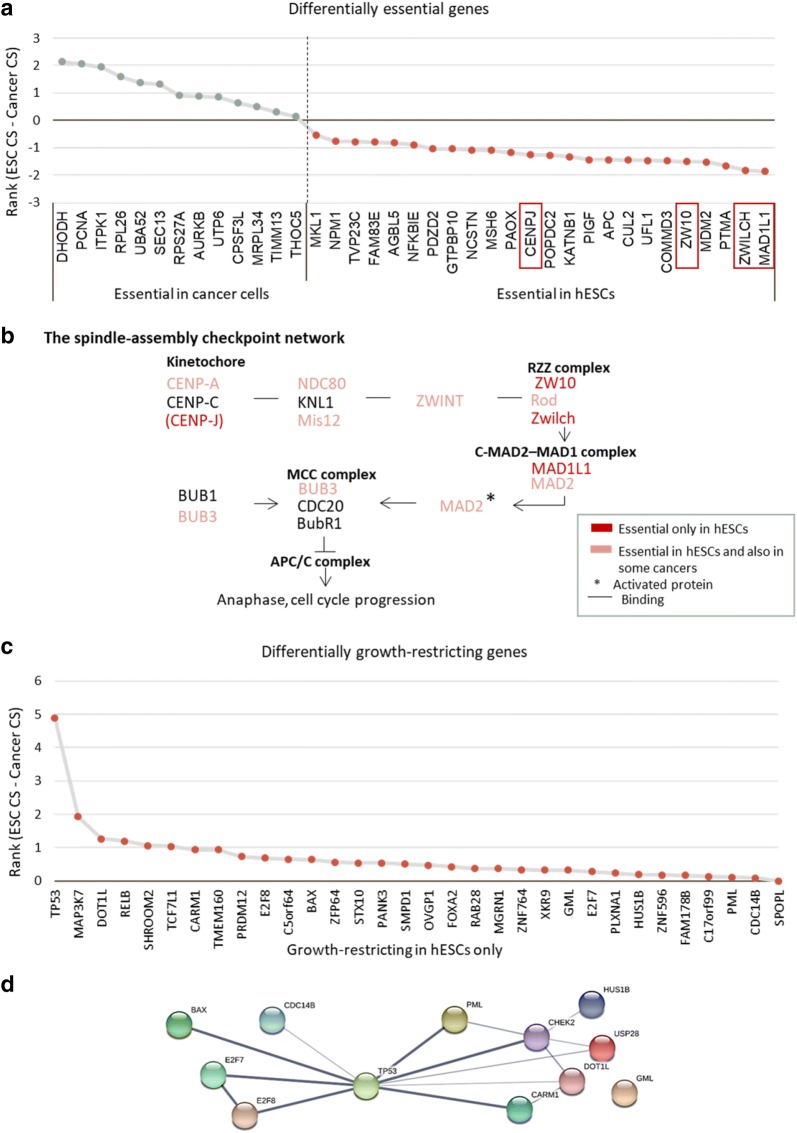
Differential essentiality and growth-restriction analysis of cell cycle genes in ESCs and cancer cells: **a** Differentially essential genes ranked according to a differential score calculated by subtracting cancer cell CRISPR score of each gene from its ESC CRISPR score. Genes associated with the spindle-assembly checkpoint are highlighted. **b** Schematic representation of the spindle-assembly checkpoint pathway. **c** Differentially growth-restricting genes between ESCs and cancer cells ranked according to the difference between the CRISPR scores obtained in the screens using these cell types. **d** Protein interactome analysis of growth-restricting checkpoint genes in ESCs

To gain more insight into the differences between ESCs and cancer cells, we analysed the genes with differential pattern of essentiality between these cells. For this, we examined the genes specified as essential in all cancer cell lines, but not in ESCs, and vice versa. Overall, we identified 24 ESC-specific essential cell cycle genes and 13 cancer-specific essential cell cycle genes (Fig. 4a). These genes may account for some of the phenotypic differences in cell cycle properties between these cell types. Among the unique stem cell-essential genes stood out a group of four closely related genes involved in the spindle assembly checkpoint (Fig. 4b) [25, 26]. This checkpoint prevents aberrant segregation of chromosomes during mitosis, thus maintaining genome integrity. Interestingly, a closer look at this pathway revealed a large proportion of ESC essential genes, suggesting a special dependence of pluripotent cells on this checkpoint (Fig. 4b).

An analysis of differentially growth-restricting genes identified 33 genes that are growth-restricting only in ESCs. No growth-restricting gene was common to all cancer cell lines (Fig. 4c). Interestingly, protein interactome analysis of the growth-restricting checkpoint genes in ESCs, using the STRING database, revealed *TP53* as the primary connecting link between these genes (Fig. 4d). *TP53* is a well-characterized tumor suppressor gene encoding the protein p53, which has a crucial role in apoptosis. *TP53* is the most frequently mutated gene in human cancers [27], and it is mutated in all the cancer cell lines examined in this study (Additional file 6: Table S5). These observations suggest that in the absence of p53 activity, a high proportion of the growth-restricting genes are not adequately regulated and thus lose their inhibitory effect. Consequently, it would take a mutation in a single gene to subvert the whole growth restriction system. Indeed, the percentage of growth-restricting genes in cancer cells is very low (Fig. 2b), and this may also be the reason for the small overlap between the growth-restricting genes in different cell lines analyzed in this study (Fig. 3c).

It is possible that the existing growth-restricting genes in cancer cells gained their effect as a result of novel mutations acquired by each cancerous cell line independently during tumorigenesis. To check this hypothesis, we retrieved the lists of background mutations acquired by each cancer cell line as documented in the Catalogue of Somatic Mutations in Cancer (COSMIC) (Additional file 6: Table S5). Interestingly, the only cell cycle gene that was mutated in all these cell lines was *TP53*. Other than that, the lists of mutant genes varied both in number and in identity, and they were associated with different pathways. These findings support our hypothesis regarding the importance of both *TP53* mutations, and the independent acquisition of random mutations in each cell line, to explain the differences in growth regulation mechanisms.

### Some key cell cycle genes are neither essential nor growth-restricting in all cell lines

Despite the central role of the checkpoint mechanisms in cell cycle regulation and the relatively high proportion of essential genes, 44.3% of the checkpoint factors did not come up as essential or as growth-restricting in all cell lines examined (Additional file 2: Table S2). Allegedly, this might suggest that these genes do not have a substantial effect on cell growth. However, an in-depth look at the identity of these genes revealed that mutations in many of them are linked to autosomal recessive disorders in humans, with severe phenotypes such as predisposition to cancer, developmental delay and neurodegeneration (Additional file 4: Fig. S4). Importantly, lack of phenotypic effect of the loss-of-function mutations in the non-essential and non-growth-restricting genes can also imply the existence of backup mechanisms that perform similar functions, thereby compensating for their absence.

### ESC-enriched cell cycle genes contain high frequency of mitosis-related genes

**Fig. 5:**
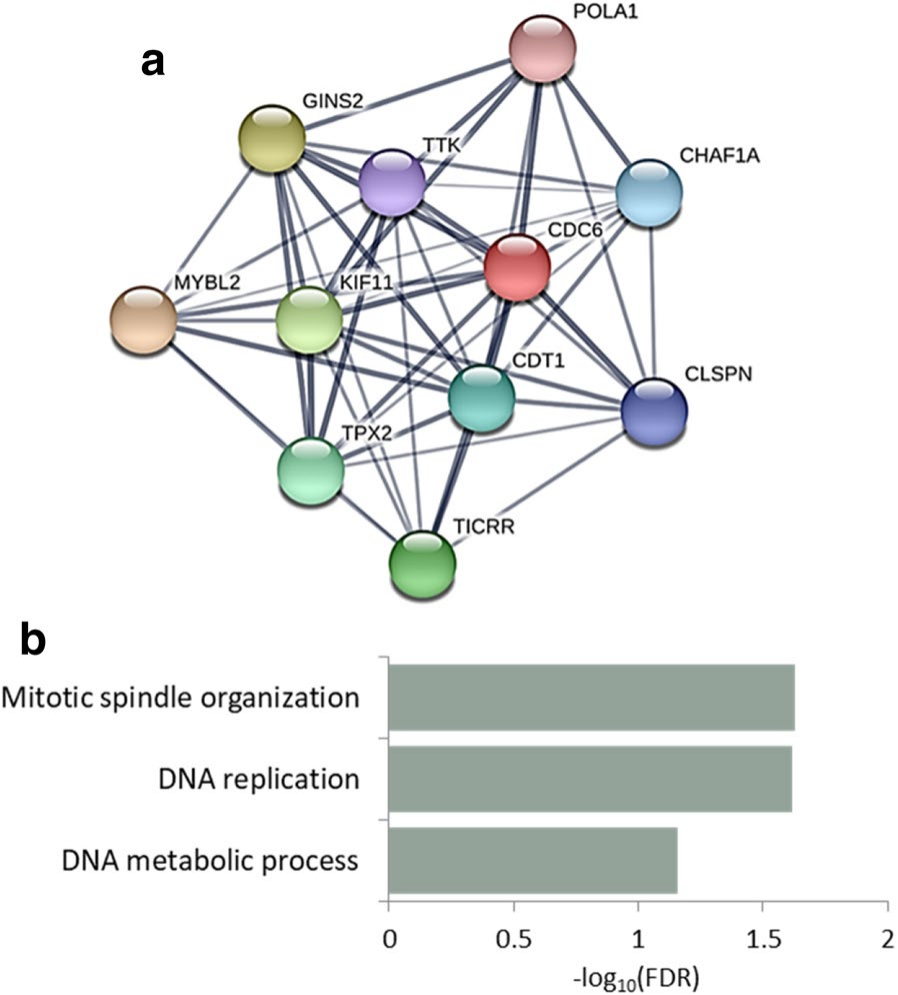
ESC-enriched essential cell cycle genes are associated with M phase checkpoint. **a** Protein interactome analysis of essential ESC-enriched cell cycle genes. **b** Gene ontology analysis of essential ESC-enriched cell cycle genes. The background used for the GO-term analysis was all cell cycle genes. Mitotic gene ontology terms are highlighted

Finally, we took an additional approach in order to identify the genetic network responsible for the ESC-specific properties. We searched for cell cycle genes which are both essential for the growth of ESCs, and are selectively enriched in expression in these cells (with expression 10 times higher in ESCs than in other tissues) [19, 20]. Notably, this analysis yielded only a small subset of genes, indicating that overall the expression of cell cycle genes is not cell type specific. However, it is interesting to note that this subset of overlapping genes constitutes a tightly connected protein network (Fig. 5a) that is highly enriched for mitotic spindle organization and DNA replication (Fig. 5b), as compared with all cell cycle genes. This is even more interesting considering our previous result regarding ESC-specific differentially essential genes (Fig. 4b), which also had a high representation of M phase checkpoint genes. Together, these results highlight the role of mitotic genes in determining the unique cell cycle characteristics of ESCs.

## Discussion

We present here a comprehensive analysis that offers a new perspective on the genetics of cell cycle regulation, based on genome-wide functional studies in cancer and pluripotent cells. Our results, while supporting established paradigms, reveal novel interesting discoveries regarding cell cycle regulation and disclose unique cell cycle features that appear in pluripotent and cancer cells.

In this analysis we defined the essentiality landscape of cell cycle genes in one haploid ESC line and four cancer cell lines. The haploid nature of the pluripotent cell line considerably improves the efficiency of obtaining loss-of-function genotypes by CRISPR/Cas9 mutagenesis and increases the chances of capturing the essentiality phenotypes. Importantly, this state of ploidy does not have a major influence on the essentialome landscape. This was demonstrated by previous studies [19, 28], and is evidenced by the fact that KBM7, the near-haploid cancer cell line, is clustered together with the other cancer cell lines rather than the haploid pluripotent cell line pES10 (Fig. 1c). Only one pluripotent cell line, pES10, was examined in this study since it was previously demonstrated that the methodological differences, such as the sgRNA library used for the screen, add noise to such comparisons [19] and no other CRISPR/Cas9 screen was performed on human ESCs using the same sgRNA library. As more screens are performed, the resolution of this analysis is expected to increase.

Given the fundamental role of the cell cycle process in the propagation of life, it is expected that the proper operation of cell cycle genes is essential for the survival and proliferation of cells, an actuality we demonstrate here. In fact, mutations in a single cell cycle gene often result in cell death or in a significant slowdown of cell growth. Intriguingly, both pluripotent and cancer cell types show higher sensitivity to mutations in checkpoint or S phase genes, compared with mutations in genes involved in other cell cycle-related processes. Moreover, the subset of S phase genes that are indispensable for cell growth is almost identical in all cell lines. This reflects the tight regulation applied on the mechanisms of DNA replication and cell cycle checkpoints, and emphasizes their importance for cell proliferation.

Notably, mutations in some of the key checkpoint factors, which regulate the activity of many downstream targets, do not have a significant impact on the growth rates of any of the tested cell lines. However, in many cases, perturbations of these genes are associated with human diseases characterized with severe consequences, including developmental syndromes with neurological symptoms. For instance, individuals with homozygous mutation in *Ataxia Telangiectasia Mutated* (*ATM*) gene, a master regulator of the DNA damage response that plays a central role in the activation of DNA damage checkpoints, were shown to develop the neurodegenerative disorder Ataxia Telangiectasia (A-T). A-T is characterized by uncoordinated movement, cancer predisposition, telangiectasia, and cerebellar atrophy [29]. Similarly, individuals with homozygous mutation in *BLM*, another central DNA damage related gene, develop the Bloom’s syndrome that is characterized by predisposition to cancer, growth deficiency and genomic instability [30]. Mutations in both *ATM* and *BLM* did not result in cell growth impairments in vitro, probably since the cells were grown in optimal conditions for a relatively short period of time. Moreover, the fact that some key cell cycle genes are not essential for cell growth may also indicate the existence of backup mechanisms that were developed through evolution to cover for the loss of function of these highly important genes. It can be interesting to try and unveil these hidden pathways by impairing more than one of these central genes simultaneously and determining synthetic lethality interactions.

Unlike the overall essentiality percentages, which were similar among different cell lines, growth restriction patterns turned out to be very heterogeneous. As a rule, cancer cells tend to have less growth-restricting genes than ESCs. Furthermore, growth restriction genes vary widely between different cancer cell lines, both in numbers and in identity. This can be explained by the central role of the tumor suppressor p53 in the regulation of the growth restriction network in normal cells. When p53 is mutated, as in the case of more than half of human tumors [27] including all the cancerous cell lines examined in this study, the growth restriction mechanism is severely disrupted. Consequently, only a few genes act as growth inhibitors in cancer cell lines. These genes acquired this role as a result of novel mutations occurred in the process of tumorigenesis. In addition, despite the similar origin of the cancer cell lines, which were all derived from the hematopoietic system, we found many differences in the CRISPR scores of cell cycle genes, reflecting the high genetic variance between human tumors. Interestingly, *TP53* was shown to be the most growth restricting gene in human ESCs [19], indicating its key role in cell cycle regulation in these cells. Moreover, *TP53* was found to be the most frequently mutated genes in human pluripotent stem cells [31], demonstrating that mutations in this gene grant a growth advantage not only in somatic cells but also in human pluripotent stem cells, and highlighting the need to ensure *TP53* integrity even when working with non-cancerous cells.

Another approach to study the different roles of cell cycle genes in cancer and pluripotent cells is to examine genes with a differential pattern of essentiality between these cell types. In this way, we have identified an increase in the essentiality of mitosis genes and of genes responsible for the regulation of tumour suppression in ESCs. In addition, several interesting individual genes have also emerged, such as *DHODH*, the highest scoring cancer-specific essential gene and *MDM2*, which is one of the highest scoring ESC-specific essential genes. *DHODH* gene encodes a rate-limiting enzyme for de novo pyrimidine nucleotide synthesis, with a role in the regulation of *VEGF* mRNA translation. *DHODH* was also shown to have a specific role in acute myeloid leukemia (AML). Inhibition of this enzyme enabled myeloid differentiation in human and mouse AML models, and it can be used as a strategy for overcoming differentiation blockade in this cancer [32]. In addition, *DHODH* inactivation or deficiency inhibits melanoma cell proliferation, induces cell cycle arrest at S phase and leads to autophagy in human melanoma cells [33]. Interestingly, in line with the known role of *DHODH*, the most dramatic effect of its knockout in this study was observed in the leukemic cell lines KBM7 and K562 (CRISPR scores − 3.4 and − 3.8, respectively), strengthening the notion for the specific role of *DHODH* in leukemia. *MDM2*, which is essential only in ESCs encodes an E3 ubiquitin ligase with proto-oncogene properties that promotes cell proliferation and tumor formation. Interestingly, *MDM2* targets p53 for degradation and thus negatively regulates p53 activity; additionally it is also transcriptionally regulated by p53 [34]. The involvement of p53 can explain the lack of phenotypic effect of *MDM2* knockout on cancer cells, which are already mutated in the *TP53* gene. As expected, the highest differentially growth-restricting gene, which affects ESCs but not cancer cells, is *TP53*. In fact, genes that have been found to be differentially essential or growth-restricting in cancer and pluripotent cells but have no established functional connection to either cell type, could also be very interesting to study. Validation and further analyses on such genes can lead to novel discoveries regarding the function of these genes in cell cycle, tumorigenesis and differentiation.

Interestingly, the unique effect of M phase genes in pluripotent cells emerged from two independent analyses in this study. Analysis of differentially essential genes determined that ESC-specific essential genes are enriched for mitotic genes. In addition, analysis of the essential cell cycle genes that are also at least 10 times more expressed in ESCs as compared with other tissues shows these genes to be highly enriched for mitotic genes. Together, these findings may imply that M phase has high essentiality in ESCs, a fact that was overlooked in previous studies. It was demonstrated that M phase in ESCs has an increased DNA repair activity [35], and that some M phase genes may play a role in the regulation of DNA repair during S phase [36]. Theoretically, such increased response to DNA damage may compensate for the short G1 that does not leave enough time for proper DNA repair [37]. This is a possible explanation for the higher abundance in essentiality of M phase genes in pluripotent cells. Yet, this is only one aspect of the entire evaluation. The distinct enrichment for genes with an established role at the spindle assembly checkpoint indicates an important role for this mechanism in the growth of pluripotent cells.

The many differences between cancer and pluripotent cells raise a serious concern regarding the frequent usage of cancer cells as a model system for cell cycle studies. Apparently, we cannot infer general conclusions regarding cell cycle regulation from cancer cells, especially concerning inhibitory pathways, which are almost absent in these cells. Of note, our analysis is partially based on a list of genes that participate in the cell cycle phases, which was retrieved from a study performed on a cancer cell line, and therefore it is probably missing some relevant genes. This emphasizes the need for functional screens performed on normal cells in order to get a more profound understanding of cell cycle genetics, and highlights the advantages of comparative studies of several cell types. In their impressive study, Mukherji et al. [21] used a series of phenotypic measurements in order to classify the cell cycle genes to the different phases. This approach is highly advantageous, but it may also lead to some classification errors, for instance in cases in which an affect in one phase is phenotypically evident only later in the cycle. One such example is BUB1 that was classified as a G1 gene, even though it is known to act during mitosis as part of the spindle assembly checkpoint (as shown in Fig. 4b). Notably, although the pluripotent cell line analyzed here is not cancerous, it is also not a normal primary cell line. In fact, ESCs share some features with cancer cells, such as unlimited capacity for cell division and fast proliferation. Thus, the differences we obtained between cancer and pluripotent cell lines may result from the stemness or un-transformed nature of pluripotent cells, and it should be taken into account while interpreting the results. That said, there are many technical limitations for the research of primary cells, such as slow growth and limited proliferation. Therefore, studying primary cells is much more challenging and demanding, and thus less common. Such studies will become easier to perform with the improvements in resolution, coverage, and costs of genetic research methods.

## Conclusions

In this study, we employed CRISPR/Cas9 libraries to explore the characteristics of cell cycle regulation in pluripotent and cancer cells. We found that genes that take part in the S phase and in checkpoint mechanisms are particularly essential for the growth of both cell types. We identified the core genetic networks that are responsible for cell cycle progression and revealed genes that are uniquely required for pluripotent or cancer cells. Interestingly, as opposed to the growth-promoting networks, the growth-restricting networks were not conserved between cell lines. This appears to be because cancer cells often harbor mutations in the tumor suppressor *TP53*, which is at the center of the growth-inhibition mechanism. Finally, a unique dependency of pluripotent cells in the process of mitotic spindle checkpoint emerged from two independent analyses in this study. Overall, our results represent new insights regarding the genetics of the cell cycle and highlight the differences between normal and transformed cell types. Further, this study indicates the inaccuracies that may arise due to the use of cells that have accumulated mutations for cell cycle studies.

## Methods

### CRISPR screen data

Data analyzed in this research was obtained from two different studies, which performed CRISPR-based genome-wide loss-of-function screens targeting 18,166 protein-coding genes, in one haploid ESC line (pES10) [19] and four cancer cell lines: two T-cell-derived chronic myelogenous leukemia cell lines (KBM7 and K562) and two B-cell-derived Burkitt’s lymphoma cell lines (Raji and Jiyoye) [22]. Importantly, the analyzed studies used the same single gRNA) library and the same method to calculate the CRISPR score of each gene.

### Defining cell cycle and checkpoint genes

List of genes of cell cycle phases was retrieved from a siRNA knockdown screen, which targeted 24,373 predicted human genes in the osteosarcoma-derived cell line U2OS in order to find genes whose downregulation disrupts the progress of the cell cycle. In total, 1152 cell cycle genes were identified in this study, and were grouped into 8 different categories, based on their function and phenotypic effect [21]. Accession numbers of these genes were converted by us to gene symbols and Ensembl IDs using the BiomaRt package with the R software. Subsequently, the gene list was filtered to include only known protein-coding sequences with up-to-date Ensembl IDs (excluding predicted mRNA models, non-coding RNAs, incomplete sequences etc.), reducing the list from 1152 to a total of 826 genes. Lastly, each gene was assigned to one of four groups, based on the phase of the cell cycle that it was shown to regulate: G1, S, S+G2 or G2/M (Additional file 1: Table S1).

List of cell cycle checkpoint genes was retrieved from the Gene Ontology database AmiGO, version 2 [38–40]. Gene names were converted to gene symbols and current Ensembl IDs using BiomaRt, leading to a total of 219 genes (Additional file 2: Table S2).

### Mutations in cancer cell lines

Lists of background mutations in the Raji, Jiyoye and K562 cell lines were retrieved from the Cell Lines Project of the Catalogue Of Somatic Mutations In Cancer (COSMIC). Mutations in KBM7 were obtained from Bürckstümmer et al. [41]. Synonymous mutations were removed, and only mutations reported in COSMIC database were chosen for further analysis. Ensembl transcript IDs were retrieved using Biomart (Additional file 6: Table S5).

### Data analysis

Lists for genes of cell cycle phases and checkpoint genes were matched with the CRISPR data to form a joint dataset of the CRISPR scores of cell cycle genes. Genes with negative CRISPR scores and with significance values (FDR or adjusted p-value) lower than 0.05 were considered as essential for cell growth. Genes with the same significance levels but with positive CRISPR scores were considered as growth-restricting. This data was used to identify cell cycle genes that are involved in the mechanisms of growth-promotion and restriction in all cell lines. CRISPR scores of cell cycle genes were compared between the different cell lines to identify common and unique cell cycle factors for cancer and pluripotent cells. STRING database of known and predicted protein-protein interactions was used for the analysis of protein interactions and identification of protein networks [24]. Functional annotation and classification of the genes were achieved using the STRING database and the GOrilla tool for identification and visualization of enriched gene ontology terms in gene lists [42, 43].

## Supplementary information

**Additional file 1: Table S1.** CRISPR scores and significance values of cell cycle phase genes, categorized by cell cycle phase.

**Additional file 2: Table S2.** CRISPR scores and significance values of checkpoint genes in all cell lines.

**Additional file 3: Table S3.** List of top 100 genes of PC1, including essentiality status.

**Additional file 4: Figure S1.** High proportion of essential and cancer-related genes in the top-100 genes that contribute to PC1. A. Fractions of essential genes out of the total cell cycle genes (grey bars) and out of the top 100 genes (red bars) in different cell lines. Asterisks represent significance level in two population proportions test. B. Gene ontology analysis for the top 100 genes. The background used for this analysis was all cell cycle genes. **Figure S2.** High overlap of essential genes across cell lines and cell cycle phases. A and B. Venn diagrams demonstrate the overlap of essential genes linked to cell cycle phases and checkpoints (A), and essential genes linked to specific phases of cell cycle among cell lines (B). **Figure S3.** Genes that are essential for all cell lines form a protein network associated with DNA replication. Interactome analysis (A) and gene ontology analysis (B) of essential genes that are common to all cell lines. The background used for this analysis was all cell cycle genes. **Figure S4.** Mutations in non-essential checkpoint genes are often associated with autosomal recessive disorders. Volcano plot demonstrating the FDR values and the CRISPR score of checkpoint genes in ESCs. Red dots indicate significantly essential (negative CRISPR score values) or growth-restricting (positive CRISPR score values) genes. Non-significant genes that are linked to autosomal recessive disorders are shown by their name.

**Additional file 5: Table S4.** List of cell cycle genes that are essential in all cell lines, S phase genes are highlighted.

**Additional file 6: Table S5.** Detailed lists of background mutations in the KBM7, K562, Jiyoye and Raji cell lines.

## Data Availability

All data analyzed during this study is available online or included in this article.
