# Supplementary material for: The essentiality landscape of cell cycle related genes in human pluripotent and cancer cells
Source: Cell Div. 2019 Dec 23;14:15. doi: 10.1186/s13008-019-0058-4 (PMC6927170; doi:10.1186/s13008-019-0058-4)
Supplement: Supplementary file 4 — Additional file 4: Figure S1. High proportion of essential and cancer-related genes in the top-100 genes that contribute to PC1. A. Fractions of essential genes out of the total cell cycle genes (grey bars) and out of the top 100 genes (red bars) in different cell lines. Asterisks represent significance level in two population proportions test. B. Gene ontology analysis for the top 100 genes. The background used for this analysis was all cell cycle genes. Figure S2. High overlap of essential genes across cell lines and cell cycle phases. A and B. Venn diagrams demonstrate the overlap of essential genes linked to cell cycle phases and checkpoints (A), and essential genes linked to specific phases of cell cycle among cell lines (B). Figure S3. Genes that are essential for all cell lines form a protein network associated with DNA replication. Interactome analysis (A) and gene ontology analysis (B) of essential genes that are common to all cell lines. The background used for this analysis was all cell cycle genes. Figure S4. Mutations in non-essential checkpoint genes are often associated with autosomal recessive disorders. Volcano plot demonstrating the FDR values and the CRISPR score of checkpoint genes in ESCs. Red dots indicate significantly essential (negative CRISPR score values) or growth-restricting (positive CRISPR score values) genes. Non-significant genes that are linked to autosomal recessive disorders are shown by their name. [file 13008_2019_58_MOESM4_ESM.pptx]

## Slide 1
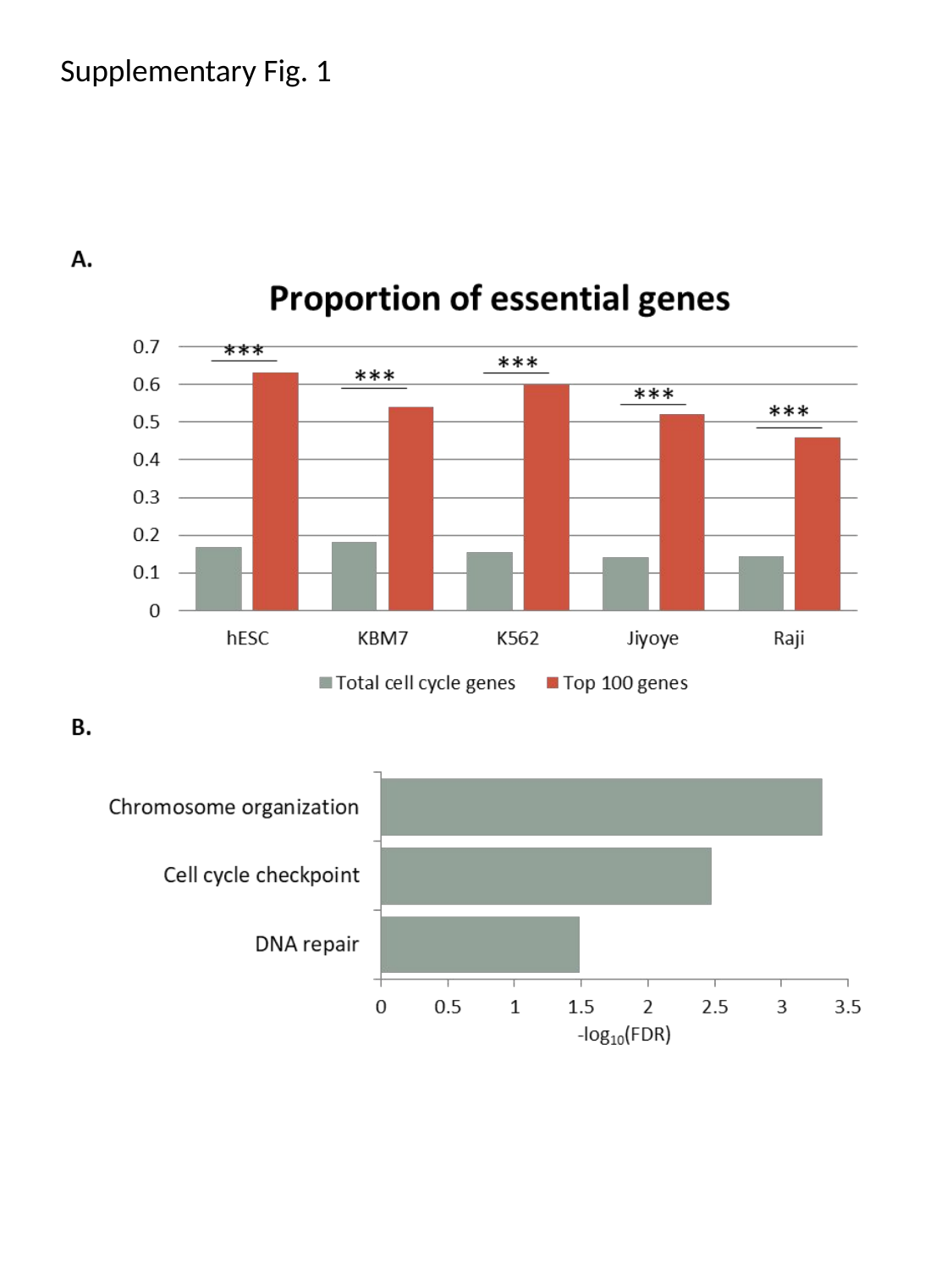

# Supplementary Fig. 1

## Slide 2
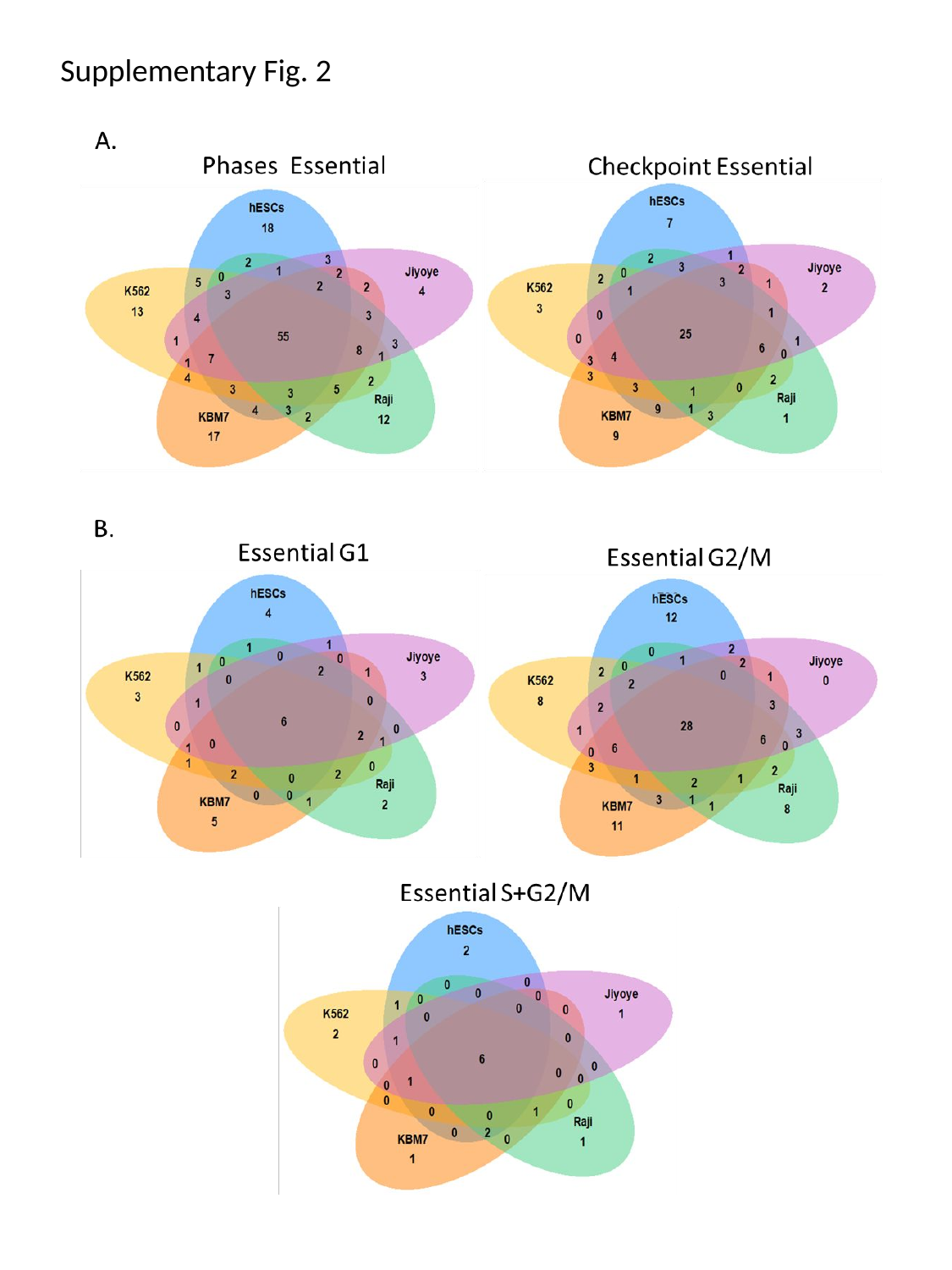

Supplementary Fig. 2

## Slide 3
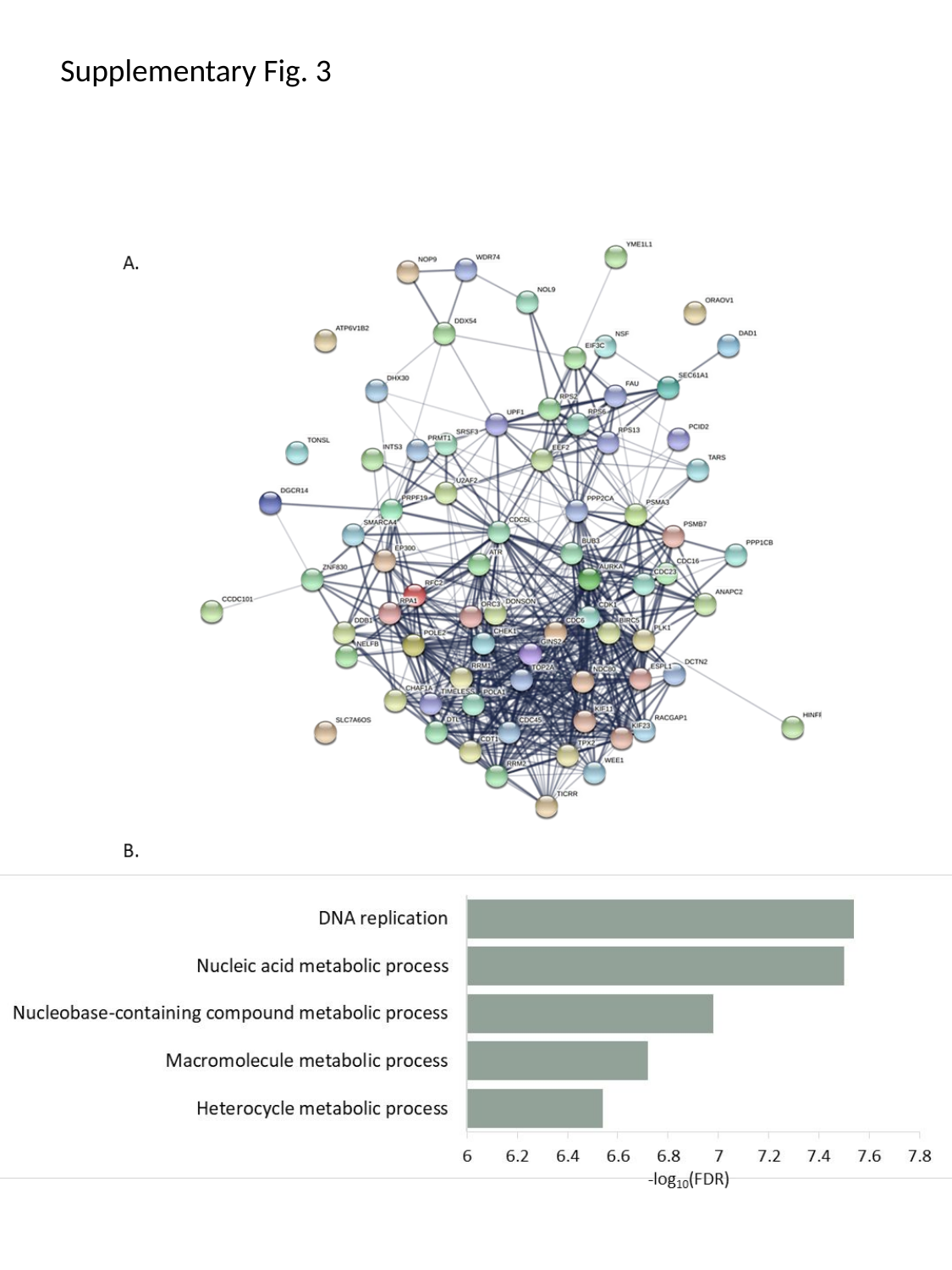

# Supplementary Fig. 3

## Slide 4
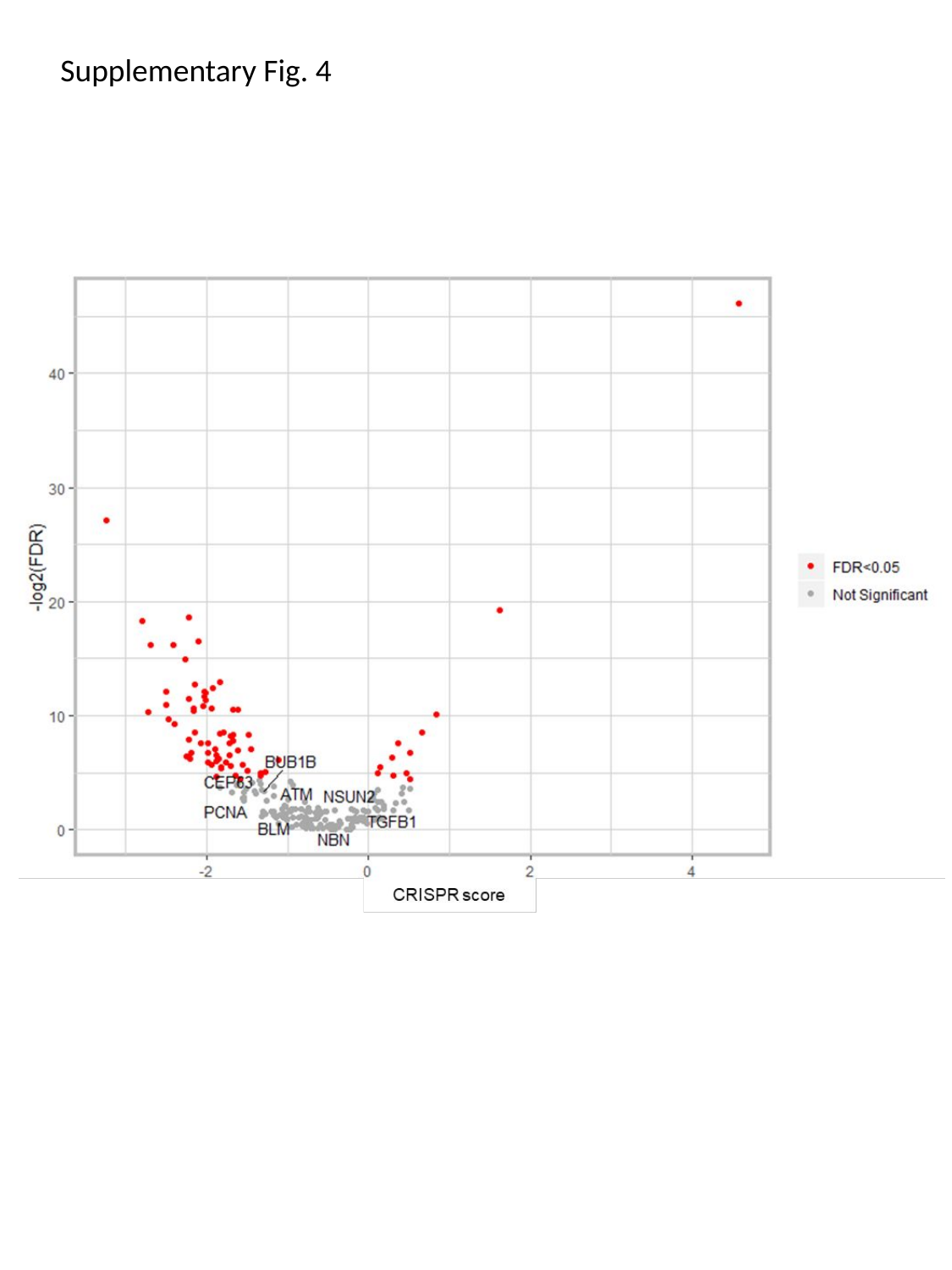

# Supplementary Fig. 4
